# Boronate ester cross-linked PVA hydrogels for the capture and H_2_O_2_-mediated release of active fluorophores†
†Electronic supplementary information (ESI) available. See DOI: 10.1039/d0cc01904f


**DOI:** 10.1039/d0cc01904f

**Published:** 2020-04-07

**Authors:** George T. Williams, Adam C. Sedgwick, Sajal Sen, Lauren Gwynne, Jordan E. Gardiner, James T. Brewster, Jennifer R. Hiscock, Tony D. James, A. Toby A. Jenkins, Jonathan L. Sessler

**Affiliations:** a Department of Chemistry , University of Bath , Bath , BA2 7AY , UK . Email: t.d.james@bath.ac.uk ; Email: a.t.a.jenkins@bath.ac.uk; b School of Physical Sciences , University of Kent , Canterbury , CT2 7NH , UK . Email: J.R.Hiscock@kent.ac.uk; c Department of Chemistry , University of Texas at Austin , 105 E 24th street A5300 , Austin , TX 78712-1224 , USA . Email: sessler@cm.utexas.edu

## Abstract

A new set of PVA hydrogels were formed using the boronate ester fluorescent probe PF1 and the novel boronate fluorescent probe PT1 as the covalent crosslinkers. Treatment with aqueous H_2_O_2_ allowed triggered release of the fluorescent dye accompanied by complete dissolution of the hydrogel.

## Abstract 2

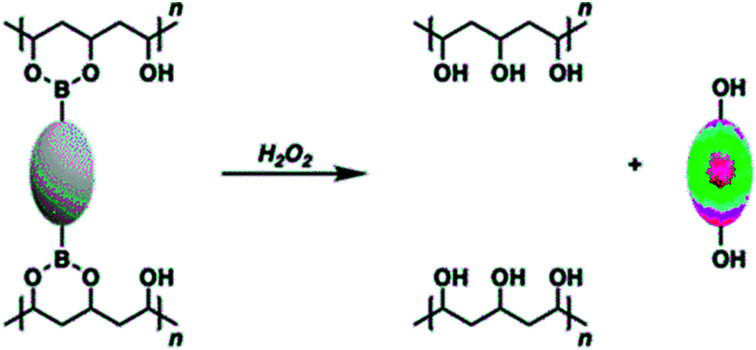
A new set of H_2_O_2_-responsive PVA hydrogels were formed using the boronate fluorescent probe **PF1** and the novel boronate fluorescent probe **PT1** as the covalent crosslinkers.

## 

Functional hydrogels have generated widespread interest as so-called intelligent devices wherein a specific stimulus can yield a macroscopic change to the self-supporting material.^1^,^2^ Such constructs offer promise in the area of drug delivery and design of “smart” wound dressings.^3^–^6^ In addition, these functional hydrogels have demonstrated great potential as fluorescent probes for live cell imaging, disease diagnosis and sensing applications with the controlled release of a fluorophore.^7^ These constructs have utilised non-covalent interactions such as aromatic–aromatic, hydrogen bonding, and hydrophobic interactions. Unfortunately, these interactions can result in the unwanted leaching of the active molecule from the hydrogel matrix. Next generation systems comprised of a pro-molecule backbone covalently linked to the hydrogel may address these issues by providing a higher local dose and sustained/controlled release of the bioactive molecule.^8^ Here, we demonstrate a new set of controlled release materials wherein hydrogen peroxide (H_2_O_2_) is used as a stimulus to release fluorophores from polyvinyl alcohol (PVA) boronate hydrogels.

Boronic acid and boronate esters have found widespread application in material-based applications, in part because of their propensity to bind reversibly with 1,2-and 1,3-diols.^9^–^19^

Such chemistry has been demonstrated *inter alia* using commercially available PVA and diboronic acid crosslinkers to afford functional PVA–boronate hydrogels.^20^–^25^ Boronic acids and boronate esters are well-known to undergo H_2_O_2_-mediated oxidative transformations to afford their corresponding phenol functionalites.^26^–^28^ We envisaged that the use of bis-boronate-based pro-molecules as cross-linkers would afford a H_2_O_2_-responsive hydrogel platform that would allow the controlled and localised release of an active molecule, such as a fluorophore (Scheme 1). It is important to note the boronate functionality is commonly used to mask active therapeutics.^29^,^30^ Currently, there is considerable interest in functionalized hydrogels wherein a specific stimulus can yield a macroscopic change to the self-supporting material, including for the stimulus-based release of specific payloads.^31^–^34^ However, new approaches to achieving such overarching objectives are still needed.

**Scheme 1:**
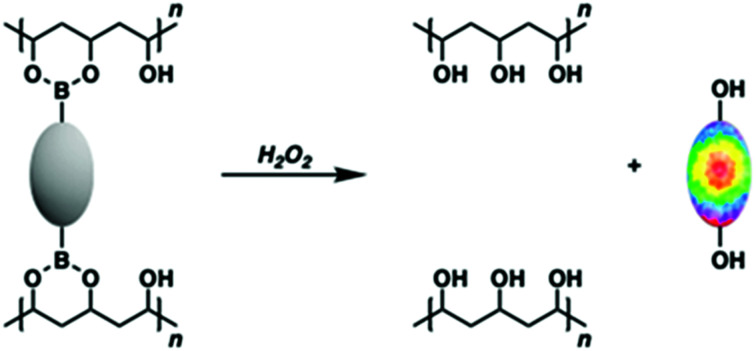
Cartoon representation illustrating the boronate pro-fluorophore encapsulated within a PVA hydrogel being activated by H_2_O_2_ to release the active fluorophore.

To address the above need, we have now prepared a new class of H_2_O_2_-responsive PVA–boronate hydrogels. These systems rely on covalent cross-linking provided solely by a set of constituent H_2_O_2_-responsive boronate ester fluorescent probes, namely the known fluorophore **PF1**^26^ and the novel fluorescent probe, **PT1** (Fig. 1). The resultant hydrogel constructs **Greenment (Gment)** and **Purplement (Pment)** displayed stability over 7 days in both aqueous solution and in the air; however, upon exposure to aqueous H_2_O_2_ the polymers were oxidised thus releasing their constituent fluorophores, fluorescein^26^ and thionol^35^ (Schemes S1 and S2, ESI†). Complete dissolution of the hydrogel could be effected depending on the specific choice of conditions as detailed below.

**Fig. 1:**
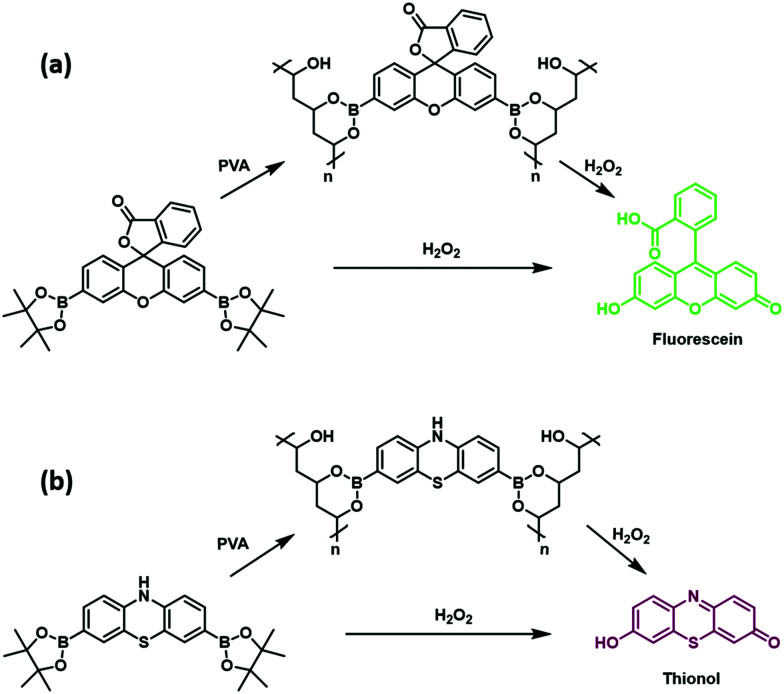
(a) H_2_O_2_-responsive fluorescent probe **PF1** and its corresponding H_2_O_2_-responsive PVA hydrogel **Gment**. (b) H_2_O_2_-responsive fluorescent probe **PT1** and its corresponding H_2_O_2_-responsive PVA hydrogel **Pment**.

**PF1** was prepared following literature procedures.^26^ The novel fluorescent probe **PT1** was synthesized through the dibromination of commercially available phenothiazine (**1**) using Br_2_ (5 equiv.) in acetic acid at room temperature, giving the desired product in 74% yield. Subsequent Suzuki–Miyaura borylation using potassium acetate, bis(pinacolato)diboron, and Pd(dppf)Cl_2_ afforded **PT1** in 43% yield.

With **PF1** and **PT1** in hand, UV and fluorescence analyses were performed. Upon exposure to aqueous H_2_O_2_ at concentrations as low as 125 μM, **PF1** exhibited a colour change from clear to green with an increase in absorption at 490 nm and an increase in fluorescence emission at 520 nm, which corresponded to the release of fluorescein (Fig. S1 and S2, ESI†). Whereas, exposure of **PT1** to H_2_O_2_ in an analogous manner led to a colour change from clear to purple and a concomitant increase in the absorption intensity at 595 nm and an increase in fluorescence emission at 610 nm. These optical changes reflected the release of free thionol as confirmed by high resolution mass spectrometry, Fig. S3–S5 (ESI†). It is important to note that in this work, we have focused on the use of these boronate-based hydrogels as materials whose controlled release may be triggered by H_2_O_2_. However, previous reports have demonstrated the greater reactivity of boronate-based fluorescent probes towards peroxynitrite (ONOO^–^).^16^,^36^–^39^ Therefore, it is likely that if used in cellular applications, both **PF1** and **PT1** could have a role to play in the fluorescence imaging of both ONOO^–^ and H_2_O_2_, albeit not necessarily in a species specific manner.

Next, the **Gment** and **Pment** PVA-hydrogels were prepared by mixing a solution of either **PF1** or **PT1** (100 mM) in dimethylsulfoxide (DMSO) with a DMSO solution of 10% PVA (low molecular weight; purchased commercially) in a 1 : 1 ratio. This solution was then heated to induce gelation, followed by heating at 60 °C overnight in an oven. The resultant gels were washed with hexanes to remove the displaced pinacol and water to remove excess DMSO. These self-supporting gels proved physically robust and stable in air and could be stored in aqueous media (PBS, pH 7.4) without degradation for 7 days until used Fig. S6–S8 (ESI†).

The ability of **Gment** or **Pment**-based PVA-hydrogel to release the corresponding dye in the presence of H_2_O_2_ was then evaluated. This was done by submerging the chosen hydrogel (200 ± 10 mg) in aqueous solutions containing different concentrations of H_2_O_2_. As shown in Fig. 2, exposure of **Gment** gels to H_2_O_2_ (0–1 mM) led to a dose-dependent increase in the fluorescence emission intensity. The colorimetric nature of **Gment** was then tested by placing the hydrogel (200 ± 10 mg samples) in an aqueous solution of H_2_O_2_ (1 mL, 1 mM). A change in colour from colourless to green ensued. Analysis of the UV-Vis absorption revealed an increase in two absorption peaks at 450 nm and 490 nm (Fig. S9, ESI†). The absorption peak at ∼450 nm is tentatively assigned to the release of monoboronate **PF3**^40^, while the absorption peak at 490 nm corresponds to the release of fluorescein. Based on this result, we believe that oxidation of only one boronate linkage is required to release the fluorescent cargo from the PVA-hydrogel system (Scheme S3, ESI†).

**Fig. 2:**
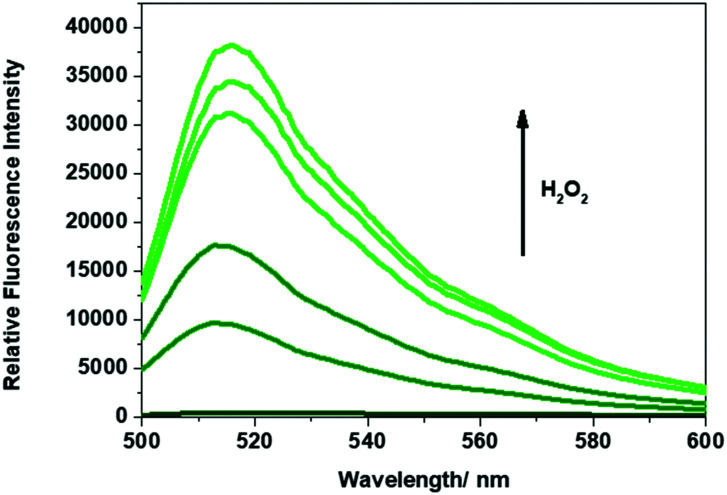
Fluorescence spectra of the supernatant of **Gment**-based PVA-hydrogels exposed to various concentrations of H_2_O_2_ (0–1 mM) in PBS, pH 7.4. Measurements were taken after 5 min at 25 °C. *λ*_ex_ = 472 (bandwith: 16 nm) on a BMG Labtech CLARIOstar® plate reader.

As shown in Fig. 3, **Pment** PVA-hydrogels exposed to various concentrations of H_2_O_2_ (0–1 mM) also led to a dose-dependent increase in the fluorescence intensity at the emission maximum of 610 nm. The colorimetric nature of **Pment** was then tested by placing the hydrogel (200 ± 10 mg samples) in an aqueous solution of H_2_O_2_ (1 mL, 1 mM). A readily discernible change in colour was observed from colourless to purple with an increase in the absorption intensity at 595 nm (see ESI† – Fig. S9–S11). In comparison to one another, **Gment** was found to be more sensitive to H_2_O_2_ than **Pment** (Fig. S12 and S13, ESI†). This finding is reflected in **Gment** having a lower Limit of Detection ((LoD) – **Gment** = 0.12 mM, LoD **Pment** = 0.33 mM). However, it is important to note, these calculated LoD values are dependent upon incubation times.

**Fig. 3:**
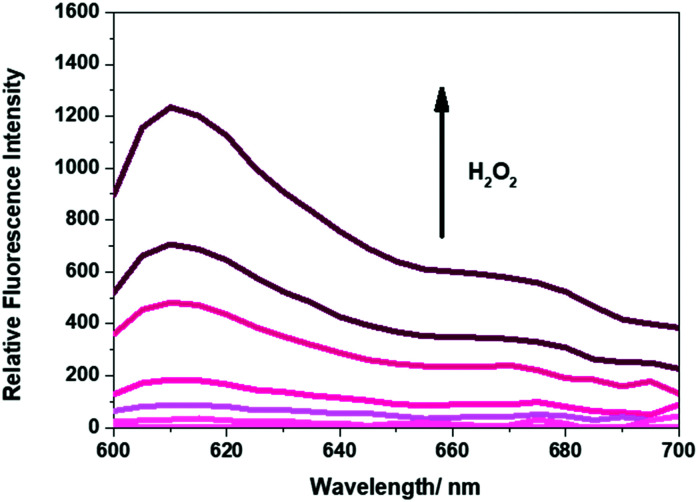
Fluorescence spectra of the supernatant of **Pment**-based PVA-hydrogels exposed to various concentrations of H_2_O_2_ (0–1 mM) in PBS, pH 7.4. Measurements were taken after 5 min at 25 °C. *λ*_ex_ = 570 (bandwith: 16 nm) on a BMG Labtech CLARIOstar® plate reader.

Notably, subjecting the hydrogels to an aqueous solution of H_2_O_2_ (100 mM) resulted in the complete dissolution of the hydrogels into solution, as shown in Fig. 4. Of note is that commercially available 3% H_2_O_2_ sold for consumer use is approximately 980 mM. The present work thus demonstrates the potential utility of boronate-based PVA polymers as a smart material for the masking and facile release of easy-to-visualise fluorophores using a readily accessible trigger. Lastly, an MTT assay with A549 cells was carried out using the **Gment** gel. At concentrations up to 50 μg mL^–1^ (note – PVA mw 13 000 – 23 000 kDa), A549 cells displayed at least 80% viability, thus demonstrating minimal acute cytotoxicity in this well-studied cell line (Fig. S14, ESI†). We believe these findings provide further support for the suggestion that the present approach may prove useful in achieving the controlled delivery of fluorescence-based diagnostics and active pharmacophores.^29^

**Fig. 4:**
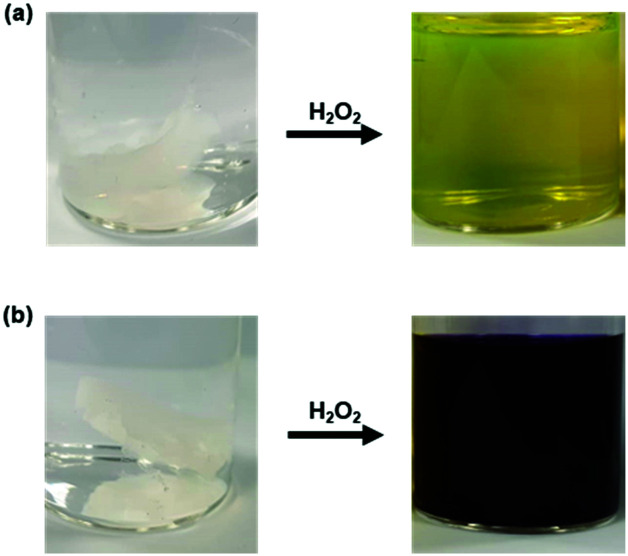
(a) **Gment** and (b) **Pment** PVA-hydrogels before and after 1.5 h exposure to 100 mM H_2_O_2_ in PBS, pH 7.4.

In conclusion, we report here the synthesis of a new H_2_O_2_-responsive bis-boronate fluorescent probe, **PT1**, and the synthesis of the previously reported H_2_O_2_-responsive fluorescent probe **PF1**. Both **PT1** and **PF1** were successfully used as diboronic acid crosslinkers to form air and aqueous stable PVA-based hydrogels. Exposure of these initially colourless and non-fluorescent systems to aqueous solutions of H_2_O_2_ allowed for the controlled release and activation of the encapsulated fluorophore. We believe these systems serve to illustrate a masking and delivery strategy that has the potential to achieve the controlled and localised release of boronic acid-based sensors and pro-drugs.^29^

The authors would like to thank the EPSRC for grant EP/R003556/1. G. T. W. would like to thank the EPSRC and Public Health England. G. T. W. and J. R. H. would like to thank the global challenges doctoral centre at the University of Kent for funding. A. C. S. and A. T. A. J. wish to thank the EPSRC for funding on smart-wound plasma – EP/R003939/1. This work was supported in part by grant MR/N0137941/1 for the GW4 BIOMED DTP, awarded to the Universities of Bath, Bristol, Cardiff and Exeter from the Medical Research Council (MRC)/UKRI. T. D. J. wishes to thank the Royal Society for a Wolfson Research Merit Award. The work in Austin was supported by the National Institutes of Health (R01 GM103790 to J. L. S.) and the Robert A. Welch Foundation (F-0018 to J. L. S.). A. C. S. would also like to acknowledge use of a Bruker AVIII HD 500 with Prodigy liquid nitrogen cryoprobe supported by NIH grant 1 S10 OD021508. All data supporting this study are provided as ESI† accompanying this paper.

## Conflicts of interest

There are no conflicts to declare.

## Supplementary Material

Supplementary information
