# Supplementary material for: Boronate ester cross-linked PVA hydrogels for the capture and H2O2-mediated release of active fluorophores
Source: Chem Commun (Camb). 2020 Apr 7;56(41):5516–9. doi: 10.1039/d0cc01904f (PMC7497407; doi:10.1039/d0cc01904f)
Supplement: Supplementary file 1 [file CC-056-D0CC01904F-s001.pdf]

Electronic Supplementary Material (ESI) for ChemComm.
This journal is © The Royal Society of Chemistry 2020

# Boronate ester cross-linked PVA hydrogels for the capture and $H_2O_2$-mediated release of active fluorophores

George T. Williams,[a,c†] Adam C. Sedgwick,[b†] Sajal Sen,[b†] Lauren Gwynne,[a] Jordan E. Gardiner,[a] James T. Brewster II,[b] Jennifer R. Hiscock,[c*] Tony D. James,[*,a] A. Toby A. Jenkins,[*,a] and Jonathan L. Sessler[*,b]

[a]Department of Chemistry, University of Bath, Bath, BA2 7AY, U.K.

[b]Department of Chemistry, The University of Texas at Austin, 105 E 24th Street A5300, Austin, TX 78712-1224, United States

[c]School of Physical Sciences, University of Kent, Canterbury, CT2 7NH, U.K.

[†]These authors contributed equally.

**Email:** t.d.james@bath.ac.uk, a.t.a.jenkins@bath.ac.uk, sessler@cm.utexas.edu, j.r.hiscock@kent.ac.uk

**Table of Contents**

1. **General Information and Protocols ........................................ 2**
2. **UV-Vis and Fluorescence Spectroscopic Analyses of PF1 and PT1 ........................ 3**
3. **HRMS Analysis of Thionol Released From Pment ........................................ 6**
4. **Stability of PVA-based Gment and Pment Hydrogels ........................................ 7**
5. **Additional Experiments for Gment and Pment Hydrogels ........................................ 9**
6. **Synthesis of Hydrogen Peroxide Responsive Crosslinkers ........................................ 13**
7. **NMR Spectra ........................................ 19**

# 1. General Information and Protocols

## General Information

All solvents and reagents were purchased from commercial suppliers and used without further purification unless otherwise noted. Analytical thin-layer chromatography (TLC) was performed using commercial precoated silica gel plates with an aluminum backing. Column chromatography was carried out using silica gel (0.040-0.063 mm). Proton and carbon NMR spectra were recorded at 298K using a Bruker Advance 500 spectrometer. Chemical shifts are reported in ppm using TMS or solvent residual signals as the internal reference standards. High-resolution mass spectrometry (HRMS) analyses were performed on an Agilent 6545 LC/Q-TOF instrument. UV-Vis spectra were recorded from 220 nm to 800 nm using a BMG Labtech CLARIOstar plate reader at room temperature using Costar U bottom 96 well microplates. Fluorescence spectra were recorded on a BMG Labtech CLARIOstar at room temperature using Greiner Bio-One microplates (Black-walled, flat bottom (chimney well), polystyrene 96-well plates).

## Gel Synthesis (Pment and Gment)

PVA (mw 13,000 – 23,000 kDa; purchased from Sigma Aldrich) was dissolved in DMSO to form a 10% w/v solution. Solutions of **PF1** and **PT1** were prepared at 100 mM in DMSO. Aliquots of the PVA solution (0.5 mL) were combined in a vial with either **PF1** or **PT1** solutions (0.5 mL). These solutions were stirred and heated with a heat gun, inducing gelation within 30 s. The gels were then heated at 60 °C overnight in an oven. The gels were subsequently washed with petroleum ether (5 mL) twice, and PBS (5 mL, pH 7.4) twice and then stored in PBS until used. These materials, based on **PF1** or **PT1**, are referred to as **Gment** and **Pment**, respectively, as noted in the main text.

## Stability studies of Gment and Pment

Hydrogels with mass 200 mg (± 10 mg) were placed in 1 mL of aqueous PBS (pH 7.4) contained in a 25 mL scintillation vial. A 100 µL aliquot of this solution was removed every day for 7 days and its UV-Vis spectrum was recorded to ensure there was no change. The 100 µL aliquot was replaced with fresh PBS to maintain a constant volume within the vial. Results indicated stability in this aqueous medium for over 7 days.

### Cell proliferation studies

A549 cells (ATCC® CCL-185™) were harvested and seeded into 96-well culture plates (Costar 07-200-90) in 100 µL of culture media. They were allowed to incubate overnight at 37 $^{o}$C in the presence of 5% $CO_2$. A549 cells were seeded at a density of 1500 cells/well. The next day, appropriate serial dilutions of drug stocks in culture media were made. To each well of a 96 well plate was added 100 µL of the appropriate solution. After a total of three days, a 50 mL aliquot of 3 mg/mL tetrazolium dye, 3-(4,5-dimethylthiazol-2-yl)-2,5-diphenyltetrazolium bromide (Alfa Aesar L11939) was added to each well, followed by a 4 h incubation period at 37 $^{o}$C. After removal of the medium, the resulting formazan was dissolved in 50 mL DMSO and the respective absorbances were measured at 560–650 nm using a microplate reader (Molecular Devices, Sunnyvale, CA). Absorbance values were corrected for background and then normalized to wells containing untreated cells to allow for plate-to-plate comparisons. Resulting dose response curves were subjected to linear regression analysis (Origin by OriginLab, Inc.) for determination of $IC_{50}$ values. The data are shown as mean inhibition of proliferation or growth as a percentage of control cells and are from 2–3 replicate experiments

## 2. UV-Vis and Fluorescence Spectroscopic Analyses of PF1 and PT1

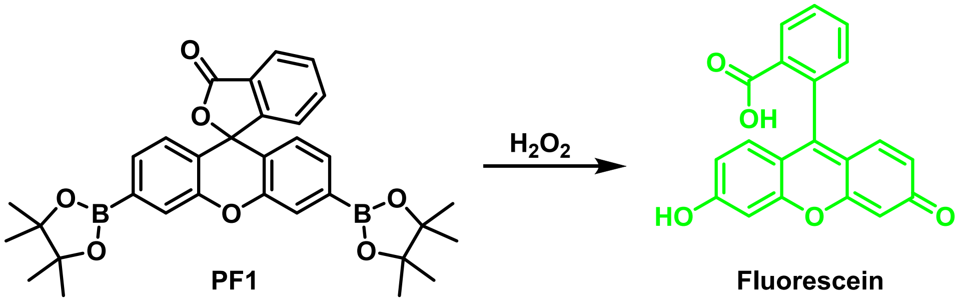


**Scheme S1 –** Aqueous $H_2O_2$-mediated transformation of **PF1** to the known fluorophore, fluorescein.

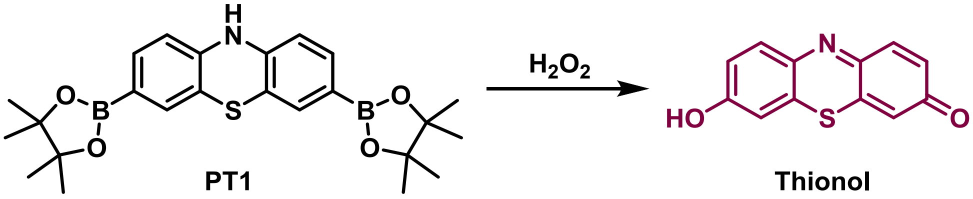


**Scheme S2 –** Aqueous $H_2O_2$-mediated transformation of the novel fluorescent probe **PT1** to the known fluorophore, thionol.

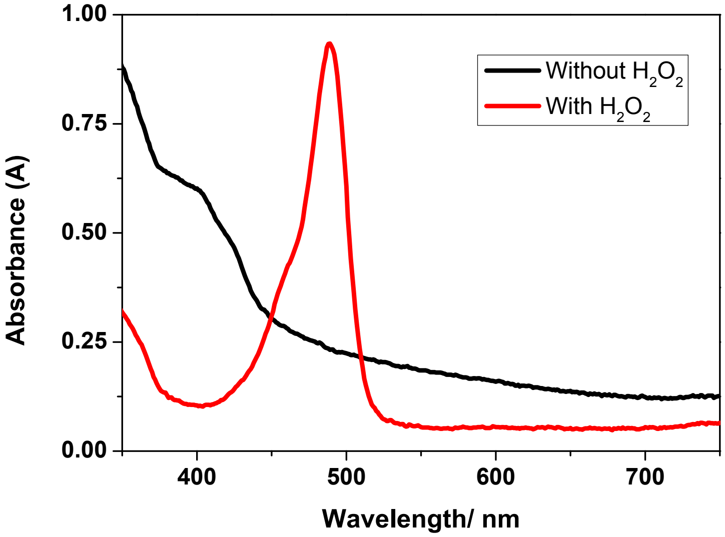


**Figure S1 –** UV-Vis spectra recorded before and after addition of aqueous $H_2O_2$ (0.5 mM) to a solution of of **PF1** (10 µM) in PBS, pH 7.4 and allowing to incubate for 10 min at 25 °C.

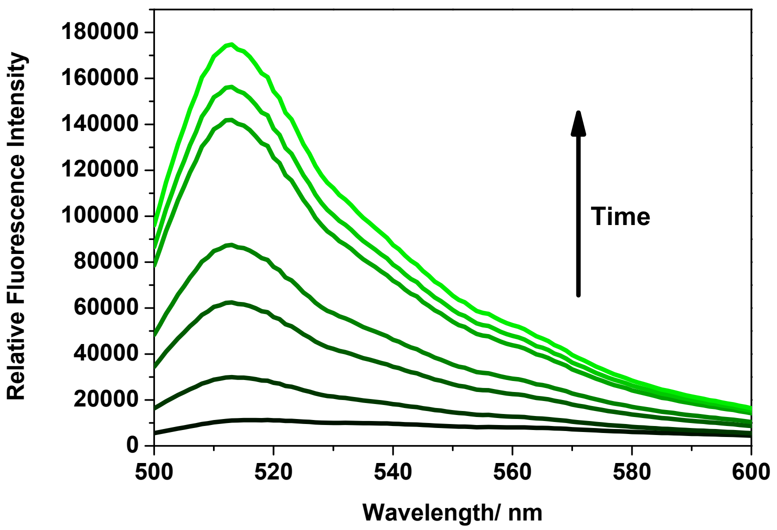


**Figure S2 -** Fluorescence spectra recorded after the addition of aqueous $H_2O_2$ (0.5 mM) to a solution of **PF1** (10 μM) in PBS, pH 7.4. Measurements were taken every 10 min over the course of 1 h at 25 °C. Arrow indicates increasing time points. $\lambda_{ex}$ = 472 (bandwidth: 16 nm).

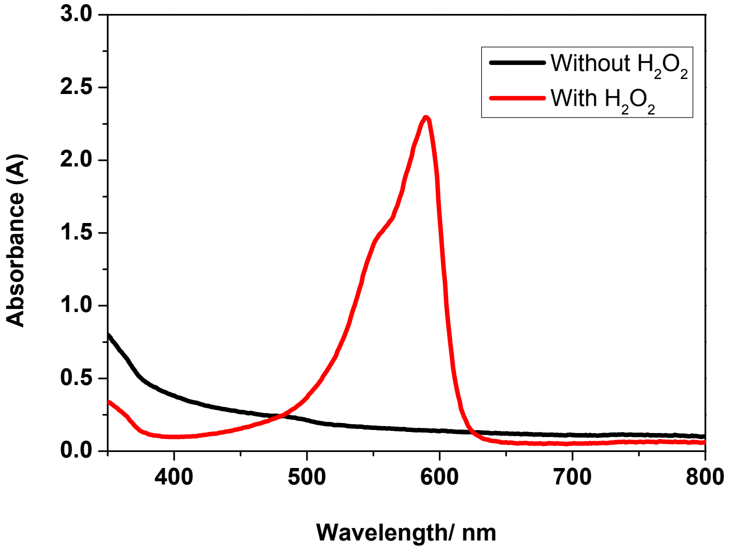


**Figure S3 -** UV-Vis spectra recorded before and after addition of aqueous $H_2O_2$ (0.5 mM) to a solution of of **PT1** (10 μM) in PBS, pH 7.4 and allowing to incubate for 10 min at 25 °C.

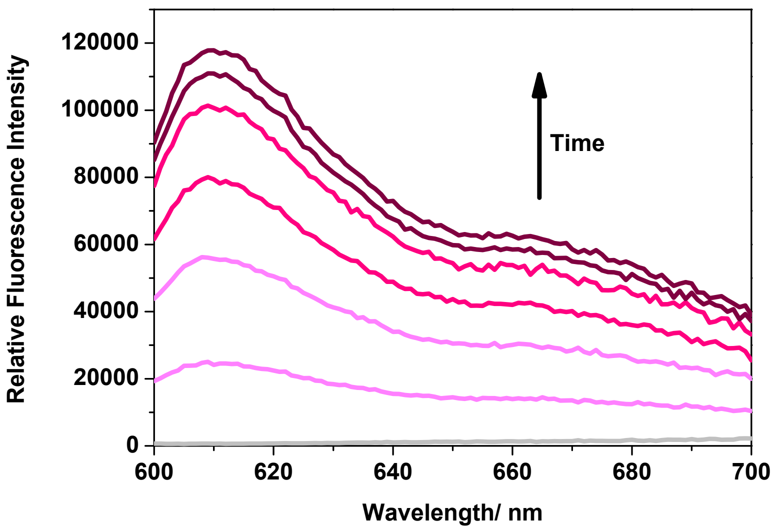


**Figure S4 -** Fluorescence spectra recorded after the addition of aqueous $H_2O_2$ (0.5 mM) to a solution of **PT1** (10 µM) in PBS, pH 7.4. Measurements were taken every 10 min over the course of 1 h at 25 °C. Arrow indicates increasing time points. $\lambda_{ex}$ = 570 (bandwidth: 16 nm).

# 3. HRMS Analysis of Thionol Released from Pment

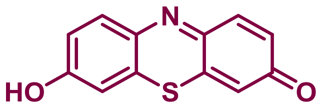


**Thionol**

**Compound Table**

| Compound Label | RT (min) | Observed mass (m/z) | Neutral observed mass (Da) | Theoretical mass (Da) | Mass error (ppm) | Isotope match score (%) |
|---|---|---|---|---|---|---|
| Cpd 1: C12 H7 N O2 S | 0.75 | 228.0125 | 229.0197 | 229.0197 | -0.22 | 98.98 |

Mass errors of between -5.00 and 5.00 ppm with isotope match scores above 60% are considered confirmation of molecular formulae

**Figure S5** – HRMS of **Pment** hydrogel treated with 1 mM $H_2O_2$ in PBS (pH 7.4) for 10 min to form thionol: m/z calculated for $C_{12}H_7NO_2S$ requires 229.0197 for $M^+$; found 229.0197.

# 4. Stability of PVA-based Gment and Pment Hydrogels

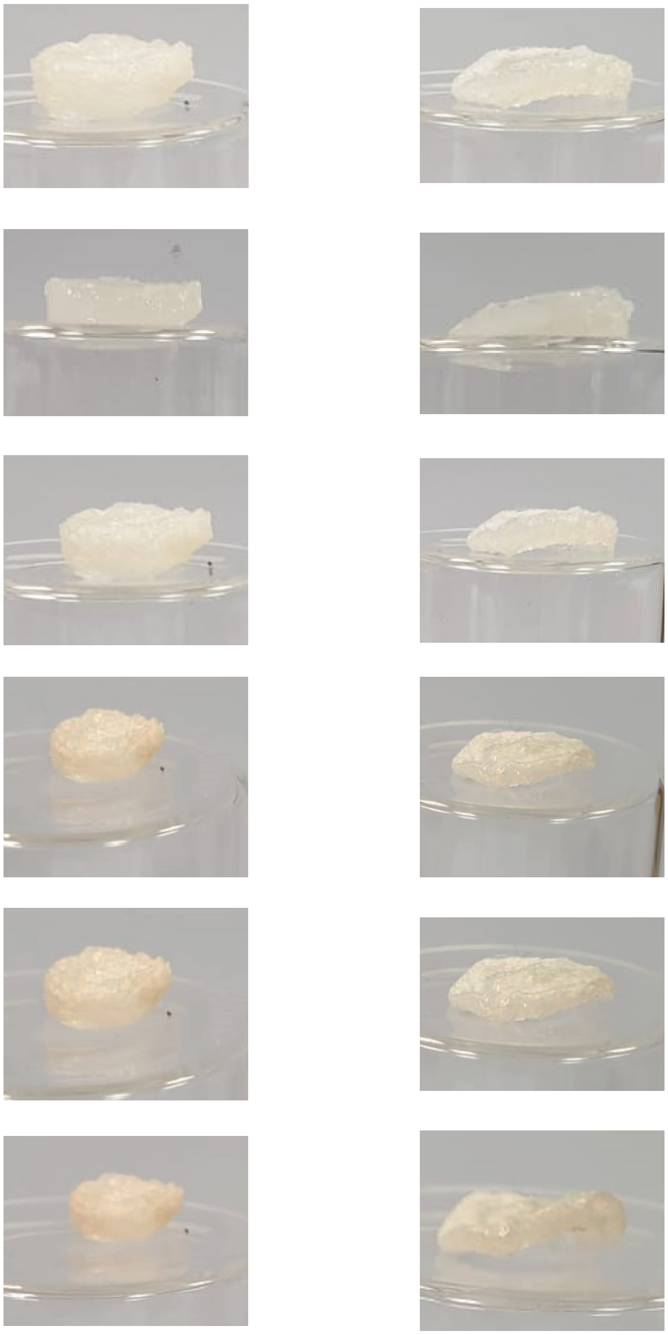


**Figure S6 –** Photographs of **Gment** and **Pment**-based PVA-hydrogels taken over the course of several days when exposed to air.

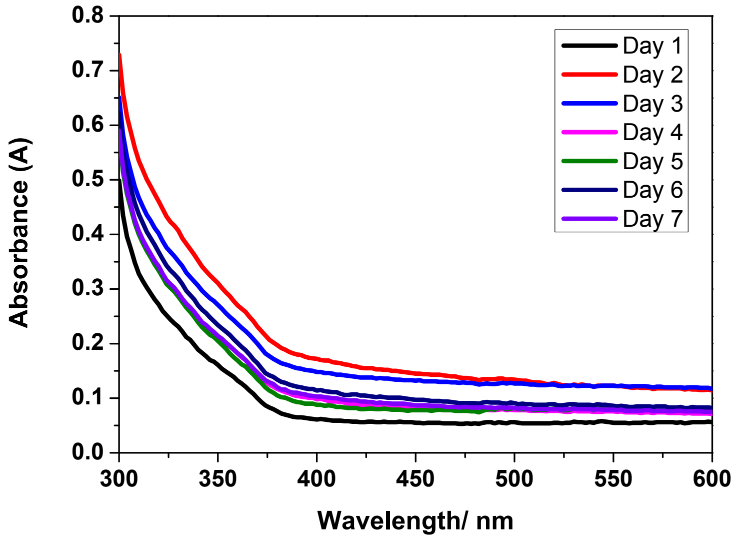


**Figure S7** – UV-Vis spectra of the supernatant of **Gment**-based PVA-hydrogels recorded over the course of 7 days while incubating in 1 mL PBS, pH 7.4 at 25 °C.

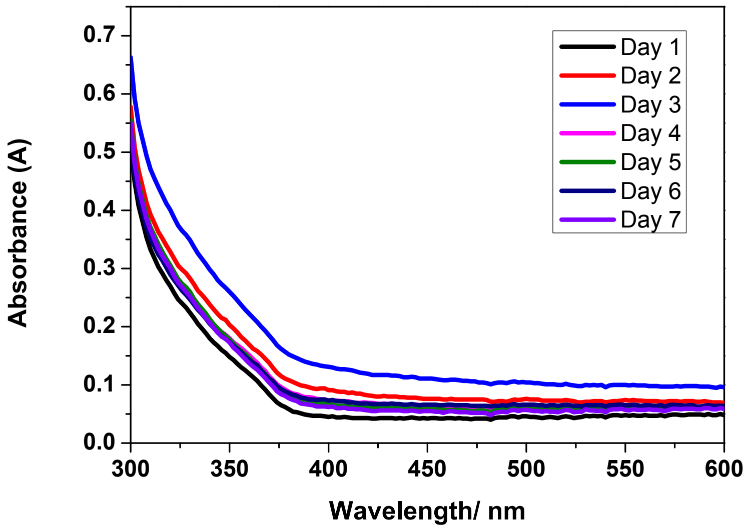


**Figure S8** - UV-Vis spectra of the supernatant of **Pment**-based PVA-hydrogels recorded over the course of 7 days while incubating in 1 mL PBS, pH 7.4 at 25 °C.

# 5. Additional Experiments for Gment and Pment Hydrogels

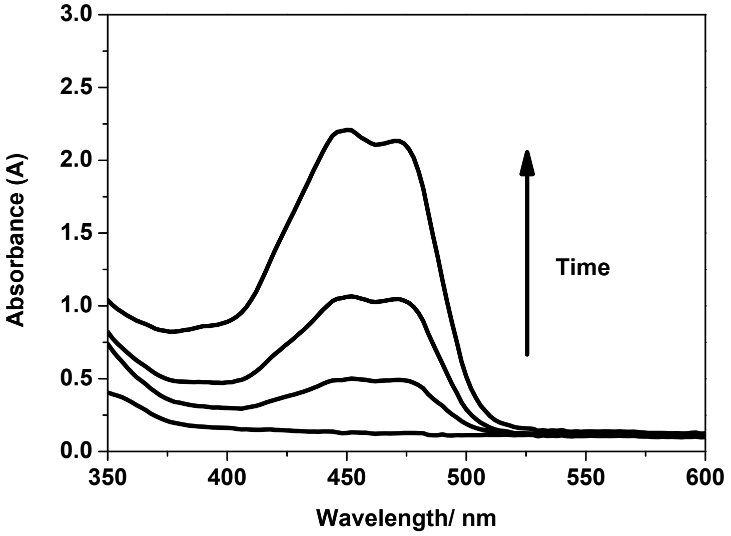


**Figure S9** - UV-Vis spectra of the supernatant of **Gment**-based PVA-hydrogels after the addition of 1 mM $H_2O_2$ in PBS, pH 7.4. Measurements were taken over the course of 90 min at 30 min intervals at 25 °C. Arrow indicates increasing time points.

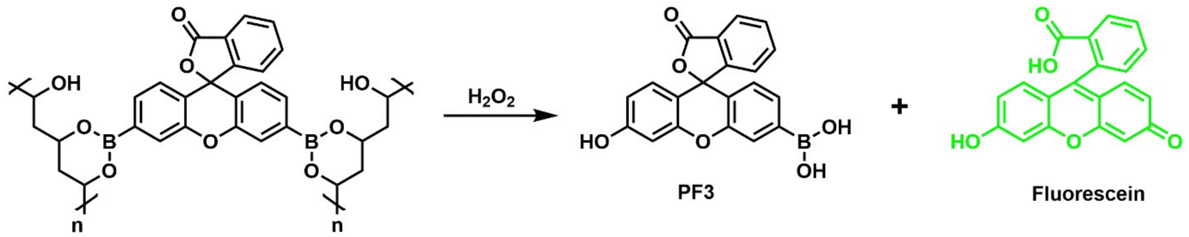


**Scheme S3 -** $H_2O_2$-responsive **Gment** gel resulting in the release of mono-boronic acid **PF3** and fluorescein.

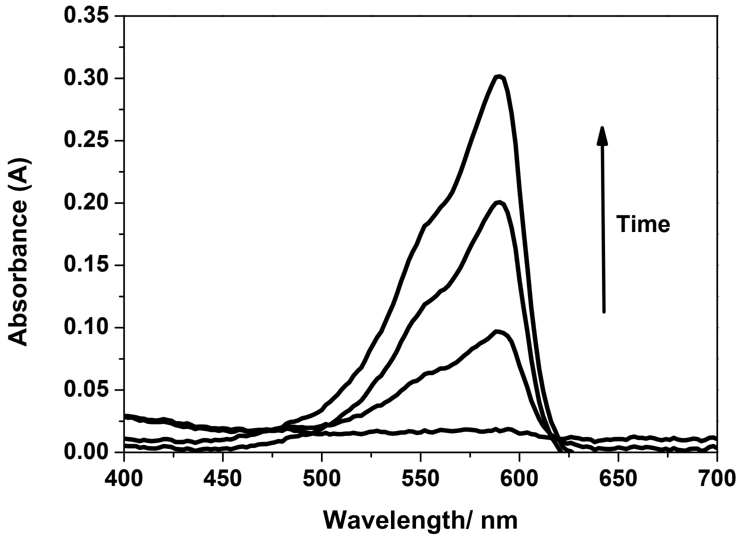


**Figure S10** - UV-Vis spectra of the supernatant of **Pment**-based PVA-hydrogels after the addition of 1 mM $H_2O_2$ in PBS, pH 7.4. Measurements were taken over the course of 90 min at 30 min intervals at 25 °C. Arrow indicates increasing time points.

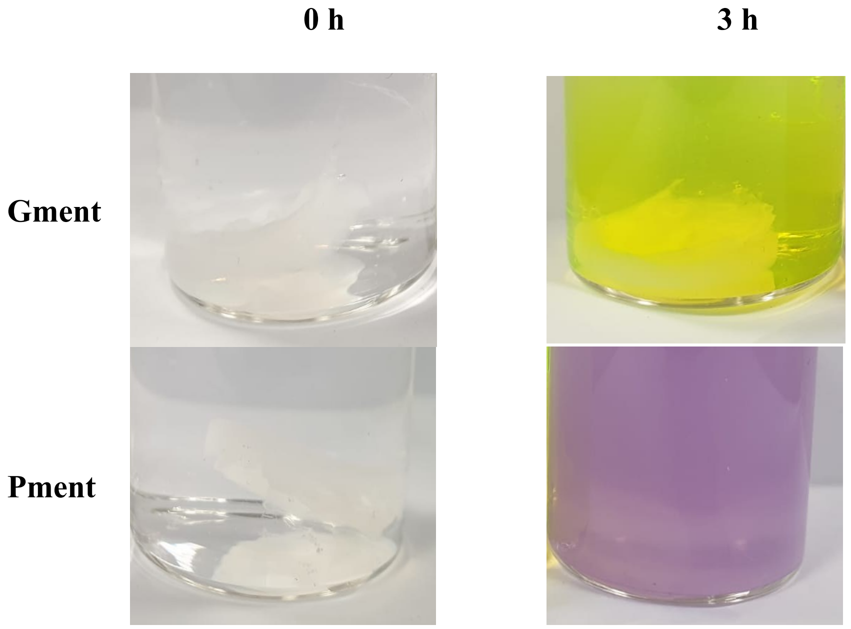


**Figure S11 – Gment** and **Pment** PVA-hydrogels before and after 3 h exposure to 1 mM $H_2O_2$ in PBS, pH 7.4.

## Limit of detection

The limit of detection was calculated using the formula shown below:

**Limit of detection (LOD) =** 3σ/slope

σ = Standard deviation of the lowest concentration

Using the graph shown below, the limit of detection for **Gment** for the detection of $H_2O_2$ at 5 minutes was calculated:

**Limit of detection (LOD) for Gment =** (3 x 4.58)/116.4 = 0.12 mM.

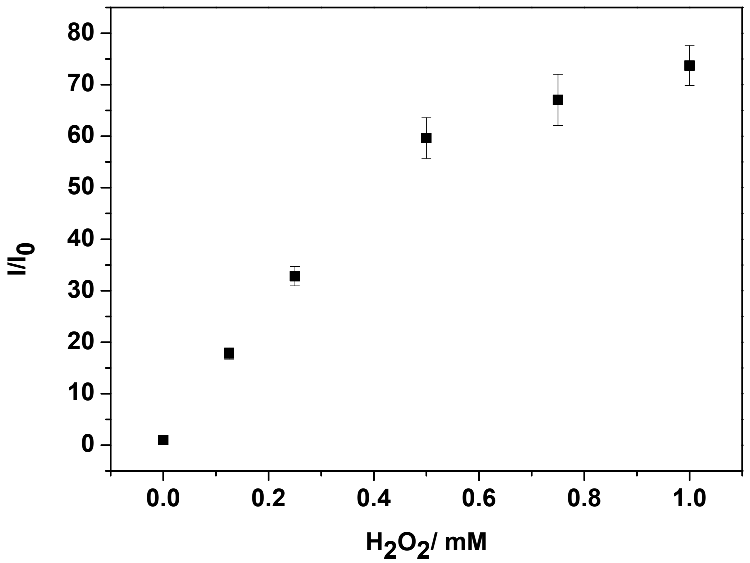


**Figure S12 –** Fluorescence intensity changes of **Gment** PVA- hydrogels exposed to various concentrations of $H_2O_2$ (0 – 1 mM) in PBS, pH 7.4. Measurements were taken after 5 min at 25 °C**.** $\lambda_{ex}$ = 472/ $\lambda_{em}$ = 520 nm (bandwith: 16 nm) on a BMG Labtech CLARIOstar® plate reader.

Using the graph shown below, the limit of detection for **Pment** for the detection of $H_2O_2$ at 5 minutes was calculated:

**Limit of detection (LOD) for Pment =** (3 x 3.42)/30.984 = 0.33 mM.

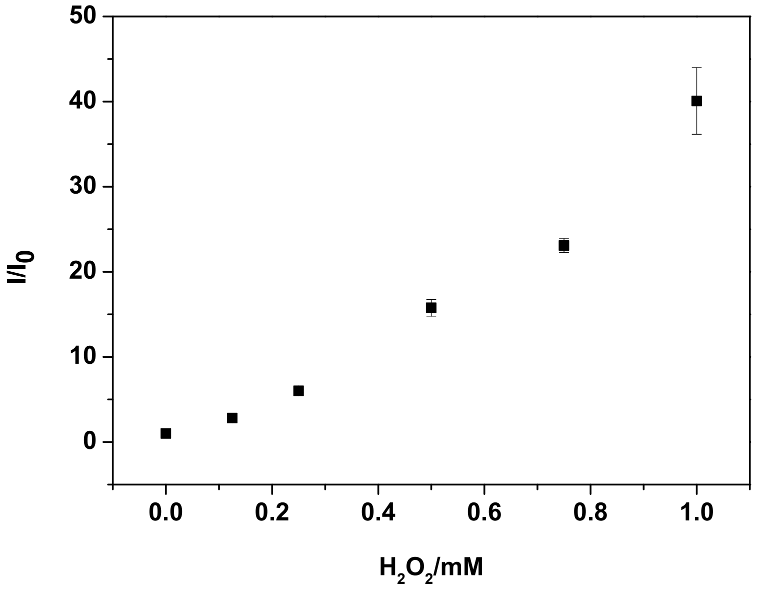


**Figure S13 –** Fluorescence intensity changes of **Pment** PVA- hydrogels exposed to various concentrations of $H_2O_2$ (0 – 1 mM) in PBS, pH 7.4. Measurements were taken after 5 min at 25 °C. $\lambda_{ex}$ = 570/ $\lambda_{em}$ = 610 nm (bandwith: 16 nm) on a BMG Labtech CLARIOstar® plate reader.

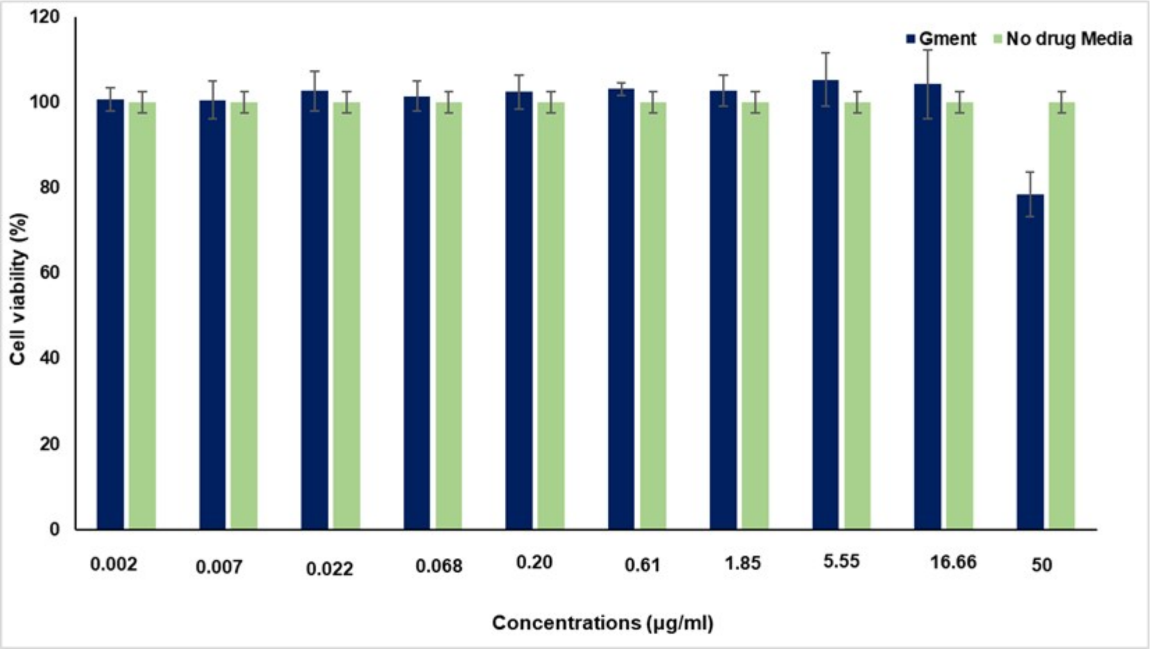


**Figure S14 -** Cell Viability of A549 cells treated with increasing concentrations of **Gment** followed by incubation for 72 h at 37 $^{o}$C.

## 6. Synthesis of Hydrogen Peroxide Responsive Crosslinkers

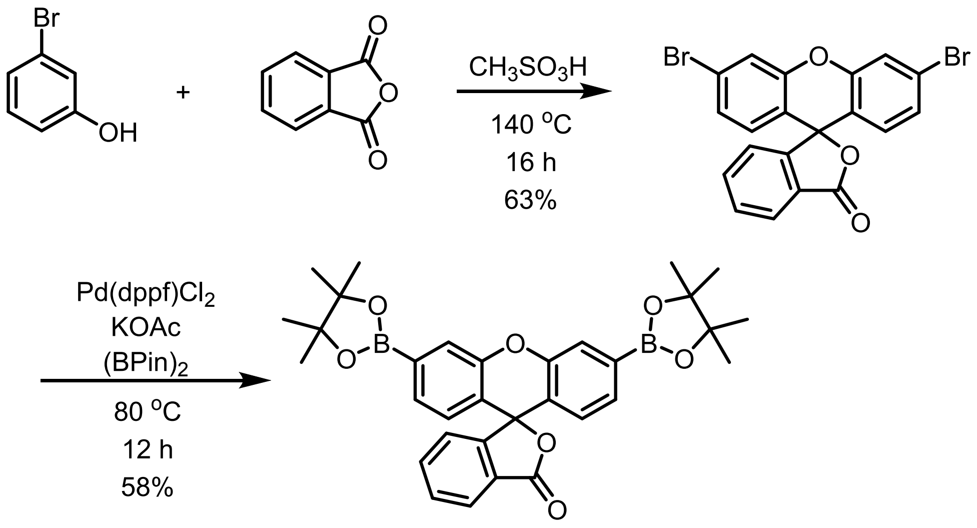


**PF1**

**Scheme S4 -** Synthetic scheme for the preparation of **PF1**.

**3',6'-Dibromo-3H-spiro[isobenzofuran-1,9'-xanthen]-3-one**

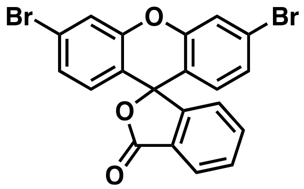


3-Bromophenol (3.66 g, 20.0 mmol) and phthalic anhydride (1.43 g, 10.0 mmol) were dissolved in methanesulfonic acid (10 mL). The reaction was stirred under reflux at 140 °C for 16 hours. The reaction was quenched with ice. The resulting reaction mixture was extracted with EtOAc (3 x 30 mL) then washed with water and sat. aqueous NaCl. The combined organic extracts were dried over $MgSO_4$ and filtered before the solvent was removed *in vacuo*. The crude product obtained in this way was purified by trituration in petroleum ether yielding a dark grey solid (2.87 g, 6.3 mmol, 63%). M.p. 289-290 °C. $^1$H NMR (δ 500 MHz, $CDCl_3$); 8.04 (1H, d, $J$ = 1.0 Hz); 7.66 (2H, m); 7.49 (2H, d, $J$ =1.9 Hz, Ar*H*), 7.19 (2H, dd, $J_1$ = 8.5 Hz, $J_2$ = 1.9 Hz, Ar*H*); 7.12 (1H, d, $J$ = 7.5 Hz, Ar*H*); 6.70 (2H, d, $J$ = 7.5 Hz, Ar*H*). $^{13}$C{$^1$H} NMR (δ 125 MHz, $CDCl_3$); 168.9, 152.7, 151.1, 135.5, 130.1, 129.2, 127.4, 125.7, 125.3, 124.1, 123.6, 120.3, 117.9. FTIR (thin film) ν max (cm$^{-1}$); 1760.91 (C=O).

**3',6'-Bis(4,4,5,5-tetramethyl-1,3,2-dioxaborolan-2-yl)-3H-spiro[isobenzofuran-1,9'-xanthen]-3-one (PF1)**

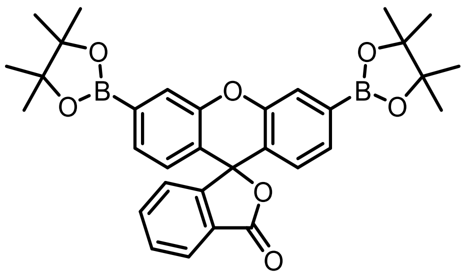


**PF1**

Dibromofluorescein (666 mg, 1.4 mmol), potassium acetate (1.30 g, 13.2 mmol), bis(pinacolato)diboron (1.33 g, 5.2 mmol) and Pd(dppf)$Cl_2$ (330 mg, 0.45 mmol) were dissolved in anhydrous degassed dimethylformamide (DMF) (5 mL) under $N_2$ in a dried 100 mL round bottomed flask. The reaction mixture was stirred at 80 °C for 12 hours. The product was extracted thrice with EtOAc and the combined organic extracts were washed three times with brine. The organic fractions were combined and dried over $MgSO_4$ and filtered, before the volatiles were removed *in vacuo*. The crude product obtained in this way was isolated as a sticky brown solid. It was purified by column chromatography (10:90 EtOAc: petroleum ether – 20:80 EtOAc: petroleum ether) yielding a pale pink solid (435 mg, 0.79 mmol, 58%). The pink colour was reduced when the solid was washed with petroleum ether. M.p. 239-241 °C. $^{1}$H NMR (δ 500 MHz, $CDCl_3$); 8.03 (1H, m); 7.73 (2H, s); 7.60 (2H, Q, $J$ = 3.5 Hz); 7.43 (2H, dd, $J_1$ = 10 Hz, $J_2$ = 1.0. Hz); 7.06 (1H, m); 6.86 (2H, d, $J$ =8.0 Hz); 1.35 (24H, s). $^{13}$C{$^{1}$H} NMR (δ 125 MHz, $CDCl_3$); 169.6, 154.0, 150.5, 135.1, 129.7, 129.3, 126.9, 125.4, 125.2, 123.6, 123.5, 121.3, 84.2, 24.7. FTIR (thin film) ν max (cm$^{-1}$); 2980.90 (sp$^3$ H), 1762.19 (C=O).

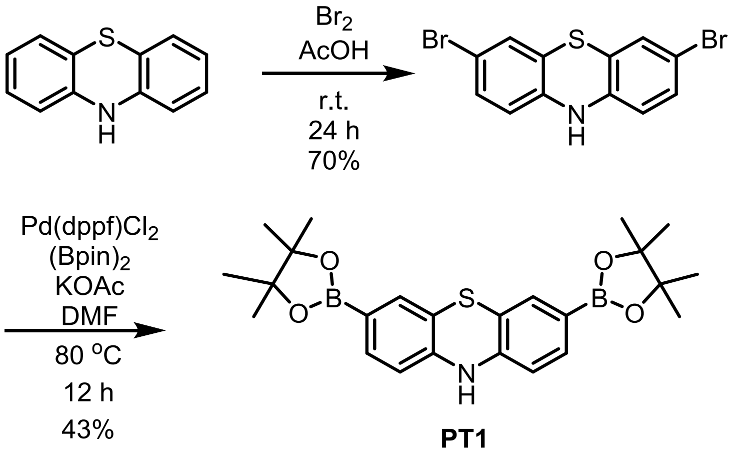


**PT1**

**Scheme S5 -** Synthetic scheme for the synthesis of **PT1.**

**3-7-Dibromo-10*H*-phenothiazine**

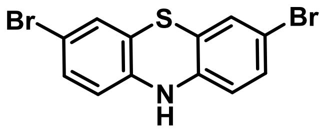

Phenothiazine (5.0 g, 25 mmol) was dissolved in AcOH (200 mL). $Br_2$ (3.3 mL, 128 mmol) was dissolved in AcOH (200 ml), this $Br_2$ solution was added to the phenothiazine solution dropwise over 1 h. The reaction was then stirred overnight at room temperature. The reaction was subsequently cooled to 0 °C before $Na_2SO_3$ (6.50 g, 51.5 mmol) was added. After stirring for one more hour, KOH (4.5 g, 80.2 mmol) in $H_2O$ (1 L) was added. The dark purple precipitate obtained in this way was isolated and recrystallized to yield the title compound as a green solid (6.60 g, 18.5 mmol, 74%). M.p. 191 – 193 °C; $^{1}$H NMR (500 MHz, DMSO-$d_6$) δ 8.85 (s, 1 H), 7.20 - 7.09 (m, 4 H), 6.58 (d, *J* = 8.3 Hz, 2 H); $^{13}$C{$^{1}$H} (125 MHz, DMSO-$d_6$) δ 141.3, 130.7, 128.5, 118.6, 116.4, 113.1; FTIR (thin film) ν max (cm-1): 3310.68 (N-H).

**3,7-Bis(4,4,5,5-tetramethyl-1,3,2-dioxaborolan-2-yl)-10H-phenothiazine (PT1)**

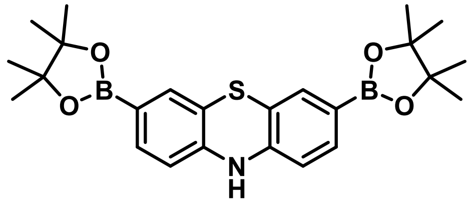

**PT1**

3,7-Dibromo-10*H*-phenothiazine (1.00 g, 2.1 mmol), potassium acetate (1.95 g, 14.7 mmol), bis(pinacolato)diboron (2.00 g, 7.8 mmol) and Pd(dppf)$Cl_2$ (500 mg, 0.68 mmol) were dissolved in anhydrous degassed DMF (8 mL) under $N_2$ in a dried 100 mL round bottomed flask. The reaction mixture was stirred at 80 °C for 12 hours. The product was extracted thrice with EtOAc and the combined organic extracts were washed three times with brine. The organic fractions were combined and dried over $MgSO_4$ before being filtered. The volatiles were removed in vacuo to give a gummy brown solid. This presumed crude product was purified by recrystallisation from dichloromethane (DCM) - MeOH to yield a yellow solid (407 mg, 0.90 mmol, 43%). $^{1}$H NMR (500 MHz, DMSO-$d_6$) δ 8.94 (s, 1 H), 7.23 (dd, J = 1.3, 10 Hz, 2 H), 7.04 (s, 2 H), 6.61 (d, *J* = 10 Hz, 2 H), 1.23 (s, 24 H); $^{13}$C{$^{1}$H} NMR (125 MHz, DMSO-$d_6$) δ 145.3, 135.6, 133.8, 118.0, 115.3, 84.7, 25.6; HRMS (FTMS-NSI): m/z calculated for $C_{24}H_{31}B_2NO_4S$ requires 450.2305 for $[M+H]^+$, found 450.2303.

# 7. NMR Spectra

**3',6'-Dibromo-3H-spiro[isobenzofuran-1,9'-xanthen]-3-one** (500 MHz, $^{1}H$ NMR, $CDCl_3$)

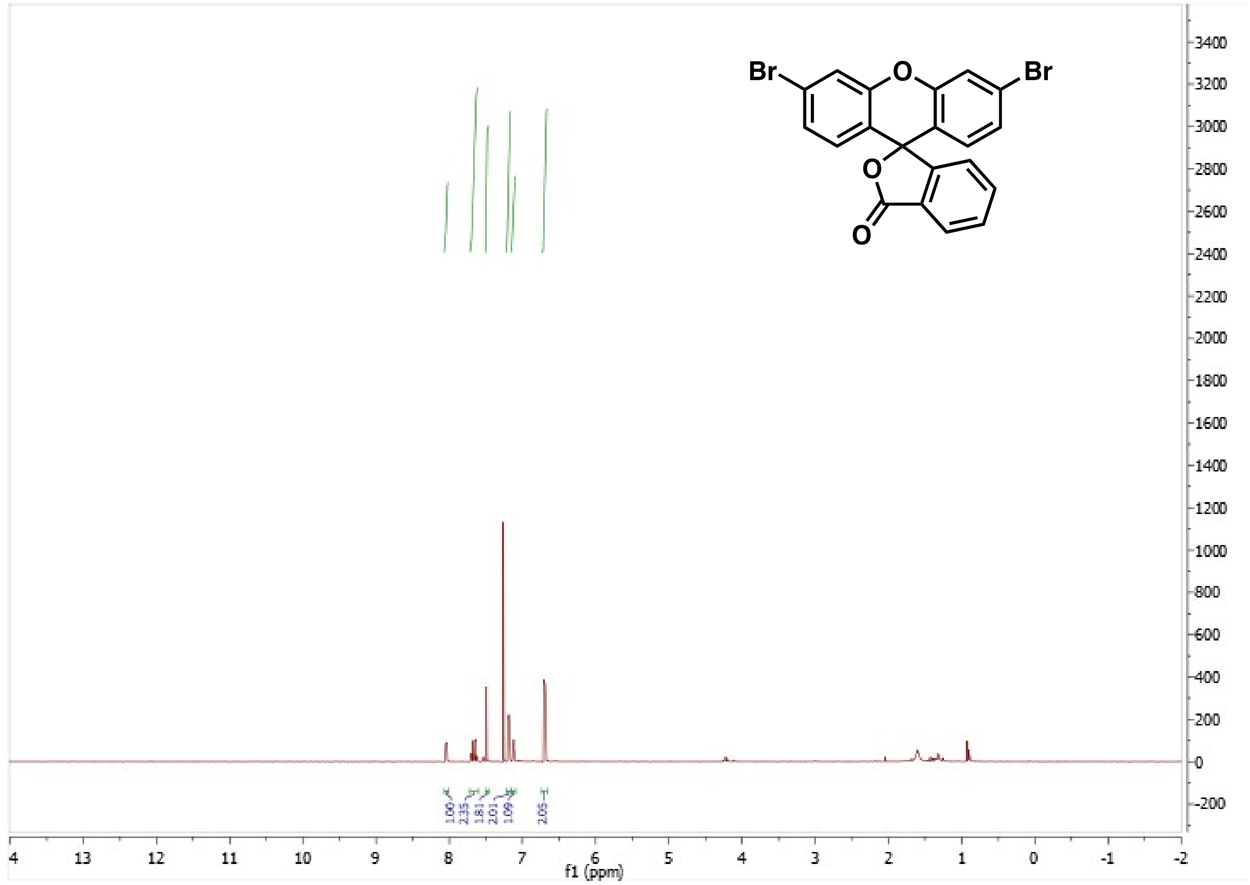

**3',6'-Dibromo-3H-spiro[isobenzofuran-1,9'-xanthen]-3-one** (126 MHz, $^{13}C\{^{1}H\}$ NMR, $CDCl_3$)

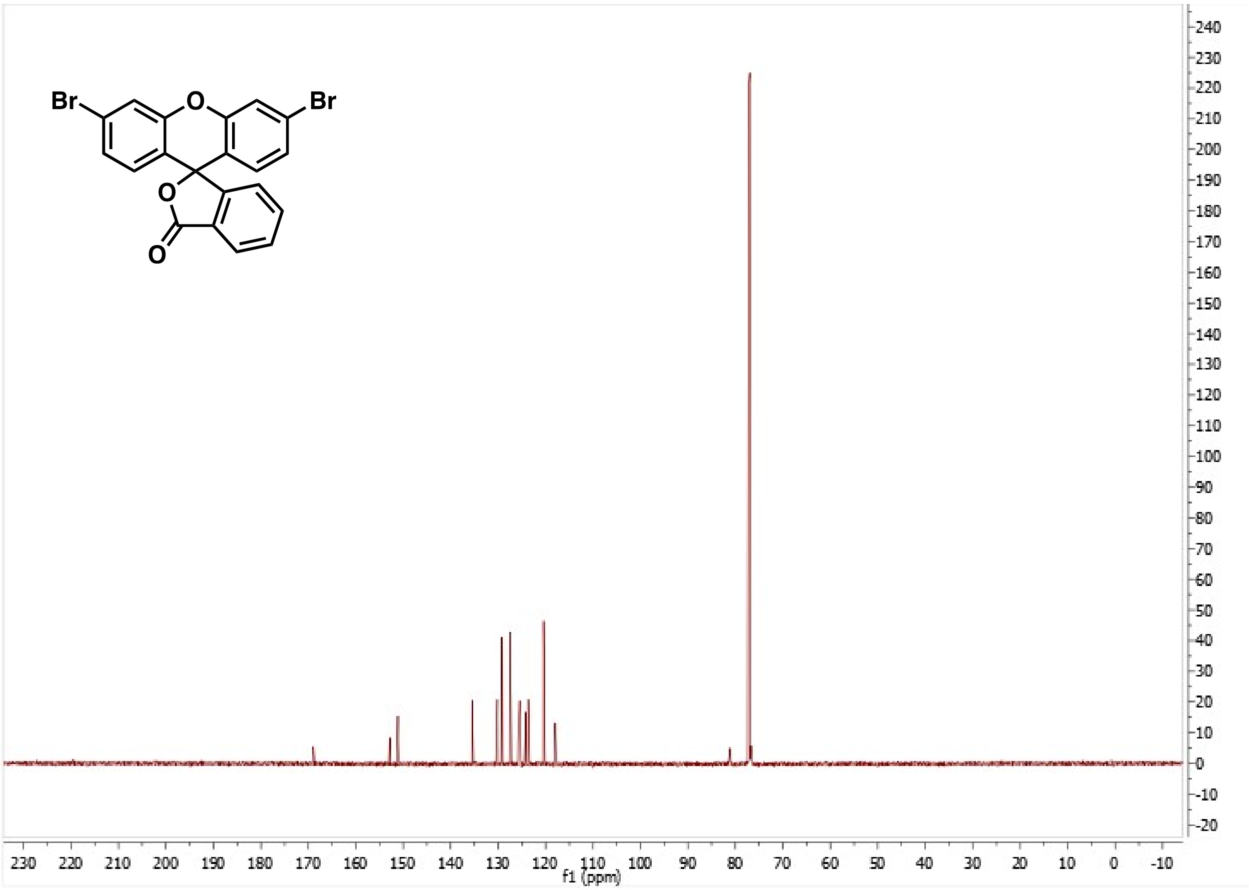

**3',6'-Bis(4,4,5,5-tetramethyl-1,3,2-dioxaborolan-2-yl)-3H-spiro[isobenzofuran-1,9'-xanthen]-3-one (PF1)** (500 MHz, $^{1}H$ NMR, $CDCl_3$)

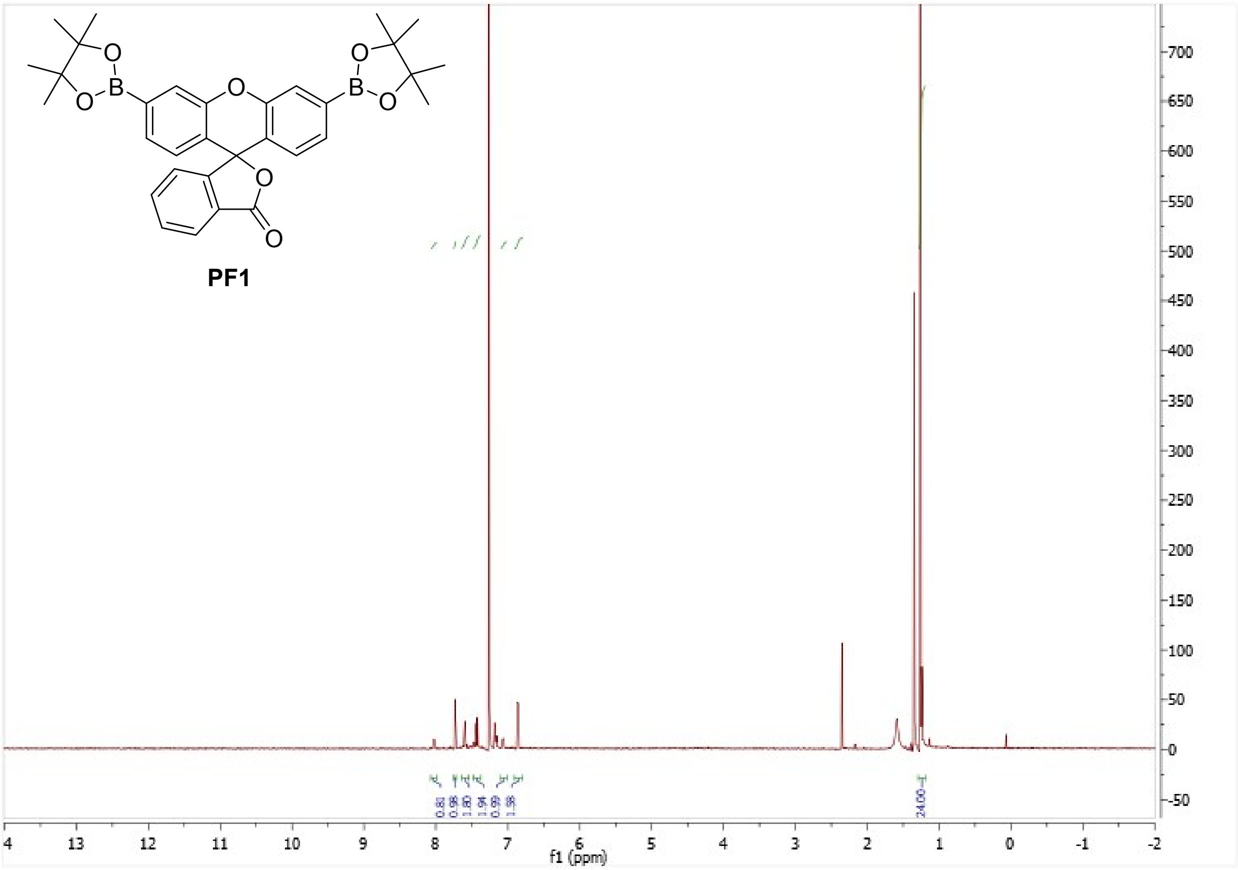

**3',6'-Bis(4,4,5,5-tetramethyl-1,3,2-dioxaborolan-2-yl)-3H-spiro[isobenzofuran-1,9'-xanthen]-3-one** (126 MHz, $^{13}C\{^1H\}$ NMR, $CDCl_3$)

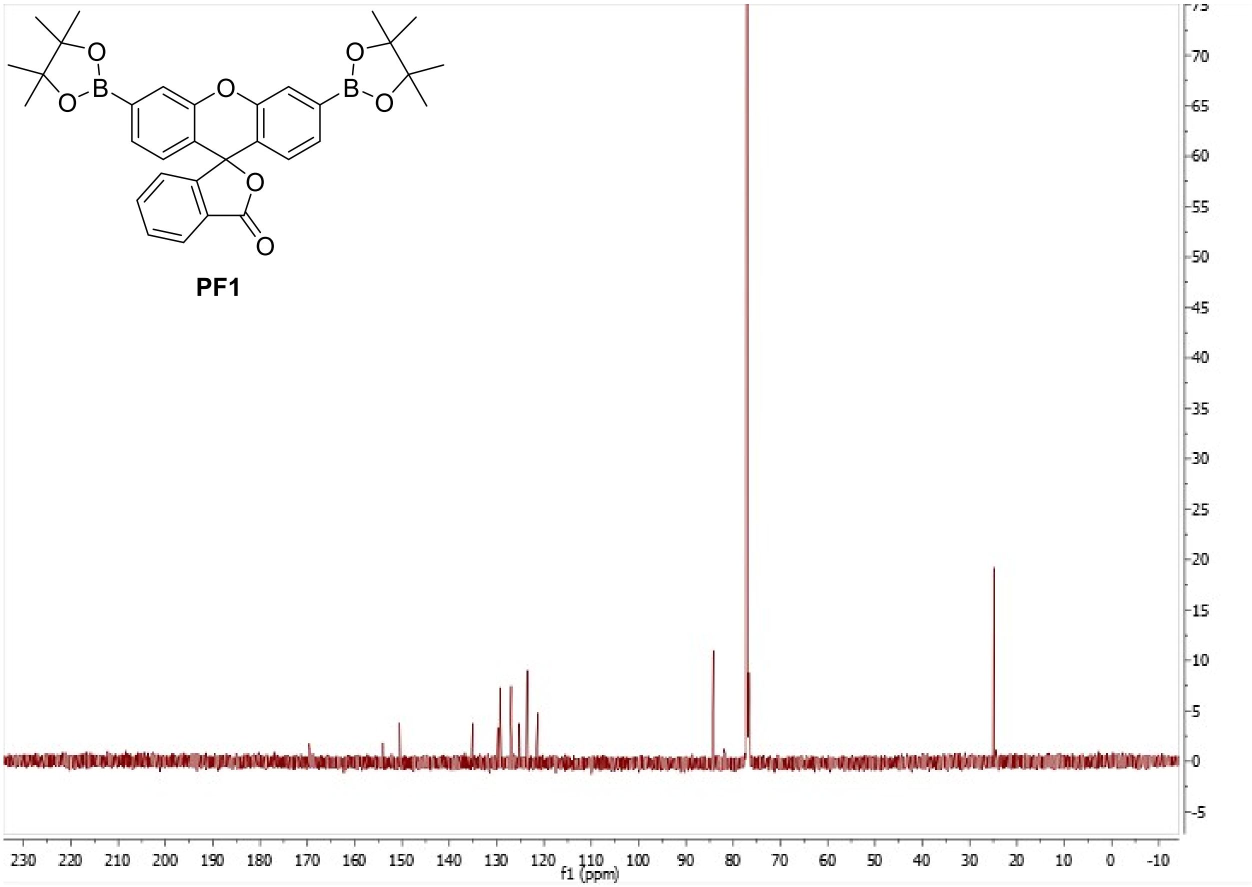

**3,7-Dibromo-10H-phenothiazine** (500 MHz, $^1H$ NMR, DMSO-$d_6$)

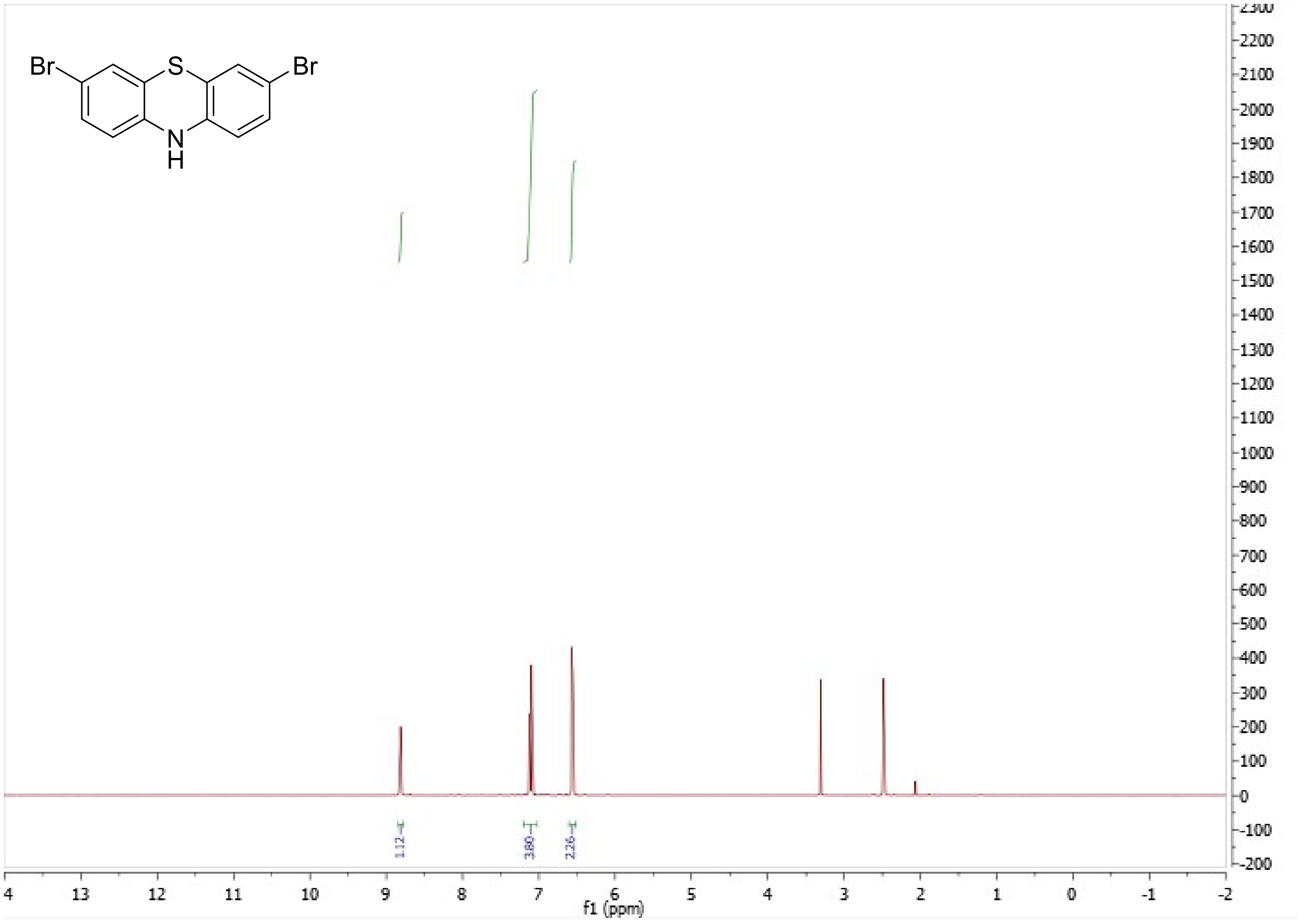

**3,7-Dibromo-10H-phenothiazine** (126 MHz, $^{13}C\{^{1}H\}$ NMR, DMSO-$d_6$)

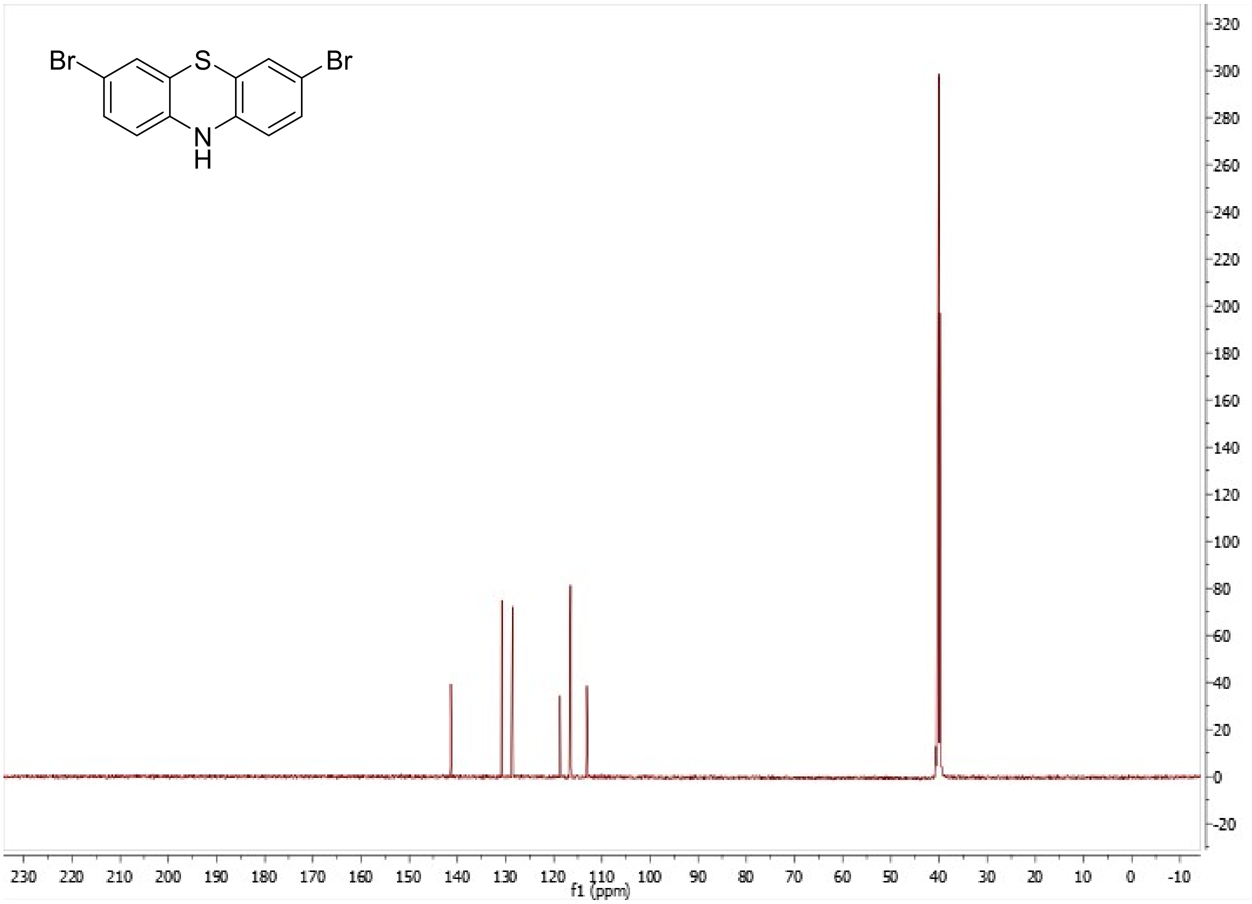

**3,7-Bis(4,4,5,5-tetramethyl-1,3,2-dioxaborolan-2-yl)-10H-phenothiazine (PT1)** (500 MHz, $^1$H NMR, DMSO-$d_6$)

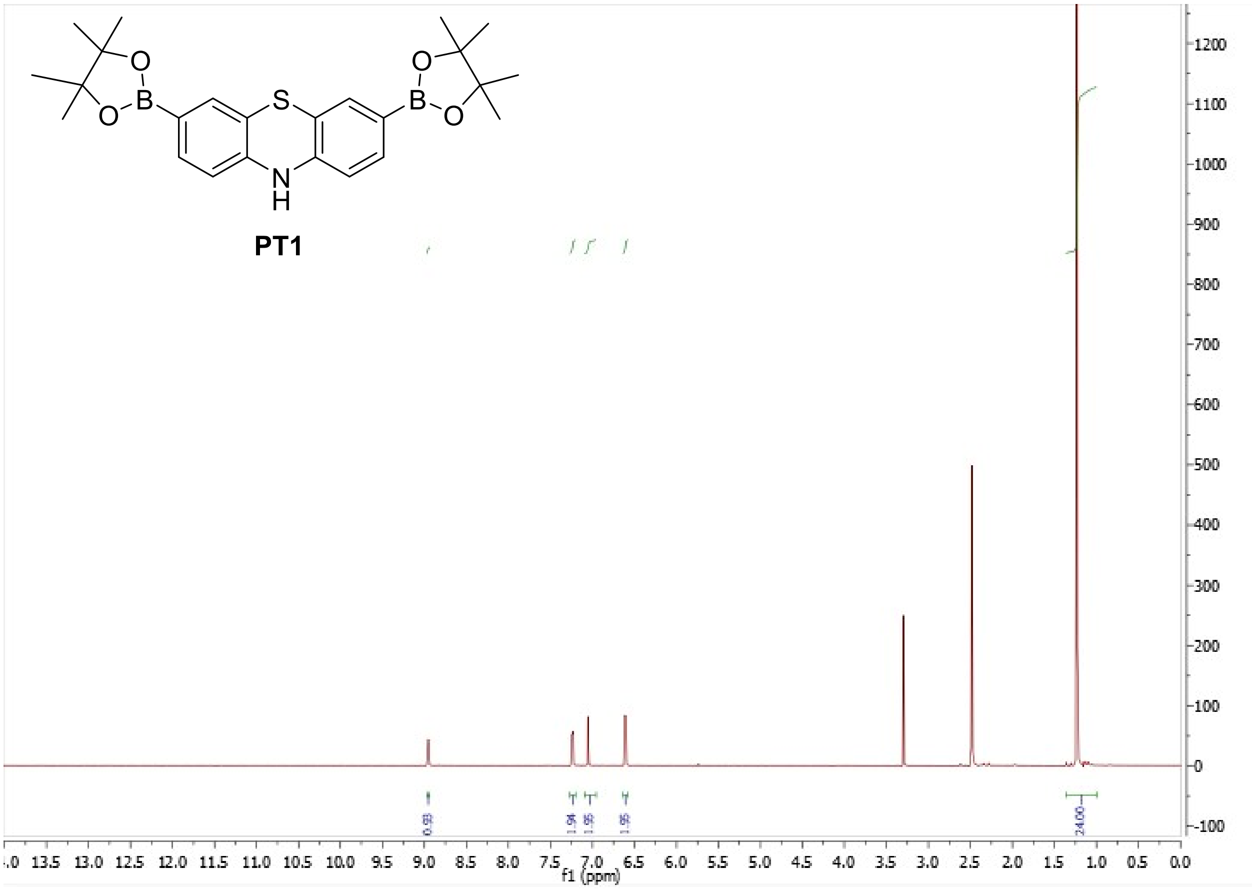

**3,7-bis(4,4,5,5-tetramethyl-1,3,2-dioxaborolan-2-yl)-10H-phenothiazine (PT1)** (125.75 MHz, $^{13}C\{^{1}H\}$ NMR, DMSO-$d_6$)

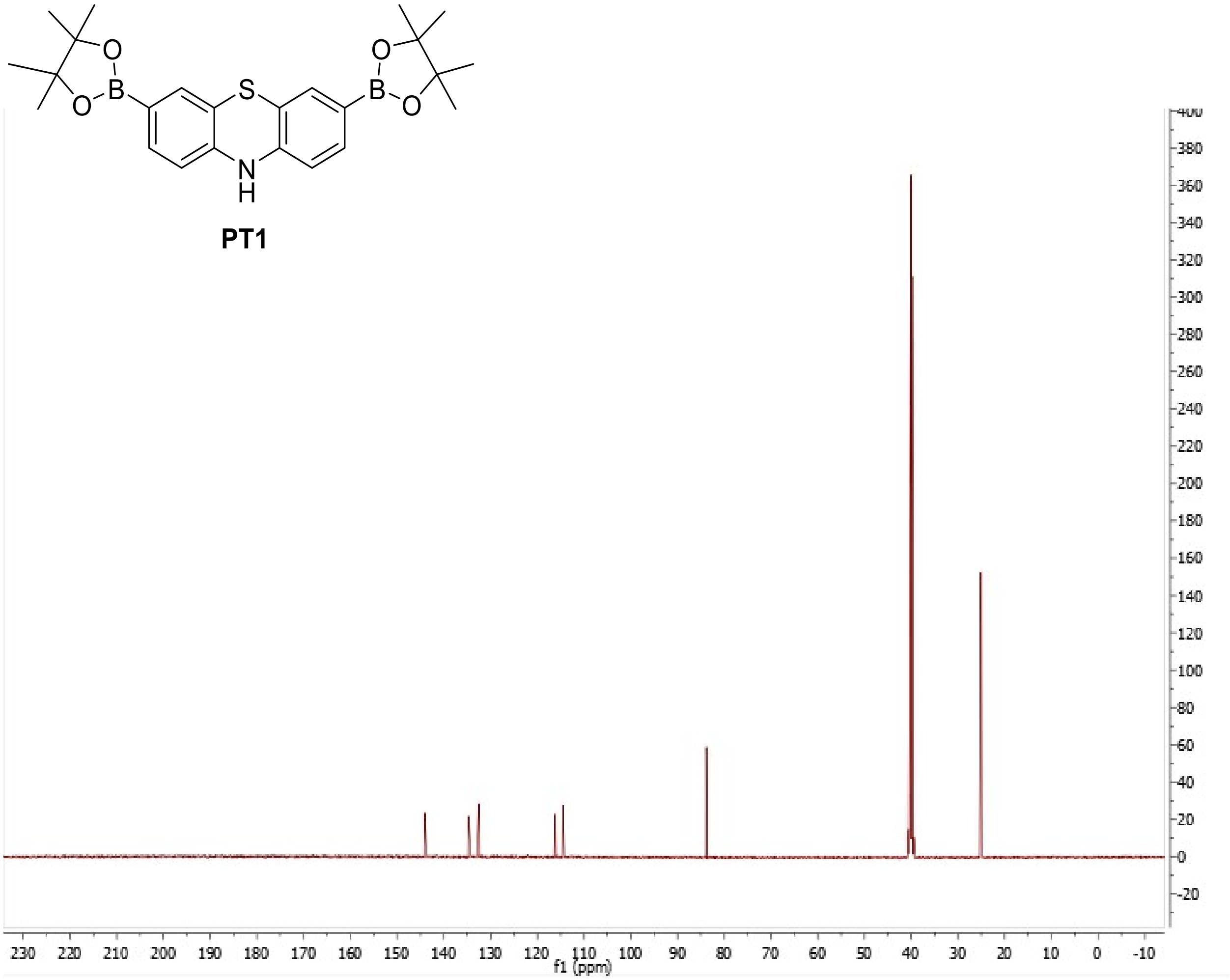


**PT1**
